# Sleepy and popular? The association between popularity, sleep duration, and insomnia in adolescents

**DOI:** 10.3389/frsle.2024.1346806

**Published:** 2024-05-01

**Authors:** Serena Bauducco, Katja Boersma, Michael Gradisar

**Affiliations:** ^1^Department of Psychology, Center for Health and Medical Psychology, Örebro University, Örebro, Sweden; ^2^College of Psychology, Flinders University, Adelaide, SA, Australia; ^3^WINK Sleep Pty Ltd, Adelaide, SA, Australia; ^4^Sleep Cycle AB, Gothenburg, Sweden

**Keywords:** social network, teenagers, sleep deficit, insomnia, peers

## Abstract

**Introduction:**

During adolescence, peers gain a central role and with the availability of technology, socializing can occur around the clock. Very few studies have focused on the role of peers in adolescents' sleep using social network analyses. These analyses describe peer relationships and social positions in a defined context (e.g., school) based on friendship nominations. Adolescents who receive many nominations can be defined as “popular,” which has been found to have its costs (i.e., shorter sleep duration) but also benefits (i.e., fewer insomnia symptoms). The aim of this study was to partially replicate and expand previous findings in a large Swedish sample of adolescents.

**Method:**

The sample included 1,394 adolescents (46% girls, *M*_age_ = 15.3, SD = 0.53, range 14–18) from 16 public schools in middle Sweden. Adolescents reported on their weekly sleep duration, insomnia symptoms, anxiety, depression, alcohol use, demographics, and nominated up to three friends in school. We used R to calculate outgoing nominations and incoming nominations. Linear regressions were used to examine the association between popularity and sleep, controlling for confounding variables (demographics, emotional problems). Finally, we explored sex differences.

**Results:**

Controlling for confounders, popular adolescents reported shorter sleep duration (B = −3.00; 95% CI [−5.77, −0.19]), and popular girls reported more insomnia symptoms (B = 0.36; 95% CI [0.04, 0.68]). There were no significant associations found for boys.

**Discussion:**

Popularity was linked to shorter sleep duration (up to −27 min for the most popular teens). Moreover, girls may pay a price for their popularity by experiencing more insomnia symptoms. Sex differences and potential mechanisms should be further explored.

## 1 Introduction

Adolescents' sleep undergoes important changes. It is increasingly recognized that sleep does not happen in isolation from the social context and the growing importance of peers is one of the hypothesized forces behind these changes (Becker et al., 2015).

The Biopsychosocial and Contextual Model of adolescents' sleep (Becker et al., 2015) illustrates the complexity of sleep during this developmental period. Biological changes predispose adolescents to develop a preference for later bed- and wake-times. These changes include a circadian rhythm delay (including, for example, a delay in melatonin onset) and a slower accumulation of sleep pressure, creating more alertness in the evening (Crowley et al., 2018). In addition, adolescents face challenges in their socioemotional sphere, including increasing school demands and worries, juggling free-time activities, gaining independence from parents (including self-selected bedtimes), and the increasing importance of peers - all of which compete with the time dedicated to sleep (Crowley et al., 2018). As a result, many adolescents experience sleep problems. Common sleep problems include insomnia symptoms (4%−39%), especially difficulties falling asleep (De Zambotti et al., 2018) and insufficient sleep duration (14%−68%) (Gariepy et al., 2020), defined as < 7 h per night (Hirshkowitz et al., 2015). While the biological and psychosocial risk and protective factors for adolescents' sleep have been widely studied (Becker et al., 2015), the peer context has received far less attention.

Both the number and quality of social connections may affect sleep. Growing evidence shows that loneliness and victimization have a negative impact on sleep quality and duration (see Gordon et al., 2021). At the other end of the continuum, having many friends and perceiving them as supportive has positive effects on sleep, but investing time in friendships might reduce their sleep opportunity on school nights (Gordon et al., 2021). Most studies rely upon adolescents' perception of social relations, whereas social network analyses help to describe peer relationships and social positions in a defined context (e.g., the school) based on friendship nominations. Based on this information, it is possible to study the complexity of the social context and how it affects people's behaviors (Neal, 2020). Peers impact adolescents' health behaviors in other risk-areas, including alcohol, drug use, and physical activity (see Montgomery et al., 2019). Yet, the impact of peer networks on sleep behaviors has received very little attention.

Only three studies to date have investigated the association between social network characteristics and sleep in adolescents (Mednick et al., 2010; Li et al., 2019; Palmer et al., 2020), and suggest that both ends of the social-connectedness spectrum were associated with impaired sleep. Adolescents with a central position within the network, or receiving many nominations (i.e., popular peers), concurrently reported shorter sleep duration (Mednick et al., 2010; Li et al., 2019) and more insomnia symptoms (Li et al., 2019). In contrast, Palmer et al. (2020) found that adolescents with fewer connections experienced worse sleep quality. Interestingly, Li et al. (2019) found that popular girls reported fewer hours of sleep, whereas lonely boys were more likely to suffer from insomnia - thus suggesting that the social context may affect boys' and girls' sleep differently. Yet, Palmer et al. (2020) did not find differences between boys and girls.

Given that the few previous studies have shown contrasting results regarding sex differences, have only included adolescents from North America, and there was variation in the quality of sleep measures used as well as sample sizes across studies, the aim of this study was to replicate the previous findings in a large Swedish sample using well-established measures of sleep duration and insomnia symptoms.

## 2 Materials and methods

### 2.1 Participants and procedure

The sample included 1,395 adolescents (46% girls, *M*_age_ = 15.3, SD = 0.5, range 14–18 years) from 16 public schools in Sweden and was drawn from a larger study (“The three cities' study”; Boersma, 2019) exploring risk/protective factors for the development of mental health problems across adolescence. The sample for this study includes grade nine students (wave 3), whose data were collected in 2016. The majority of adolescents reported living with both parents (69.6%), living with parents in separate houses (11.8%), or living with only their mother (14.4%), father (3.2%), or another guardian (1%); the majority of adolescents had a Swedish background (75%) as defined by the official Statistics Sweden (2002).

Trained test leaders administered the surveys allowing students 90 min to complete the questionnaires. Each class received 300 Swedish crowns in recognition of participation. Before participation, active consent from students and passive consent from parents were received, to increase participation rate and to limit sampling bias (Shaw et al., 2015). Moreover, students were informed about confidentiality, that participation was voluntary, and that they could choose to withdraw from the study at any time. The project was approved by the Regional Ethical Board in Uppsala, Sweden. The procedure regarding data collection is beyond the scope of this brief report, and has been thoroughly described elsewhere (Boersma, 2019).

### 2.2 Measures

#### 2.2.1 Demographics

Adolescents were asked about their sex (male/female) and age. Socioeconomic status (SES) was assessed through four questions about family affluence from the Family Affluence Scale (FAS-II) from the Health Behavior in School-aged Children (HBSC) (Boyce et al., 2006). Questions about adolescents' immigrant background included place of birth and parents' place of birth (including Sweden, Scandinavia, Europe and outside Europe). Immigrant background was defined as being born outside of Sweden or being born in Sweden with both non-Swedish parents according to Statistics Sweden (2002).

#### 2.2.2 Sleep duration

Weekday sleep duration was estimated from adolescents' self-reported bedtime (“What time do you usually go to bed on school days?”), wake-time (“What time do you usually wake up on school days?”), and sleep onset latency (“On school nights, after you go to bed, about how long does it take for you to fall asleep?”) during the past 2 weeks. These items were drawn from the School Sleep Habits Survey (Wolfson and Carskadon, 1998), which have shown good validity when compared to objective sleep measures (Wolfson et al., 2003).

#### 2.2.3 Insomnia symptoms

We used the 7-item Insomnia Severity Index (ISI) to measure symptoms of insomnia among adolescents (Morin, 1993). Items cover difficulties falling asleep, staying asleep and waking up too early, as well as perceived satisfaction with sleep, interference with daytime functioning and worry about sleep. The time frame in this study was changed from 2-weeks (in the original) to the last 6 months to match how other somatic complaints were measured in the rest of the survey. Response options ranged from 0 to 4, with higher scores indicating more severe problems and a possible total score of 28. The scale has been shown to be reliable and valid (Chung et al., 2011). In the present study, Cronbach's alpha was 0.87.

#### 2.2.4 Social network variables

**Figure 1:**
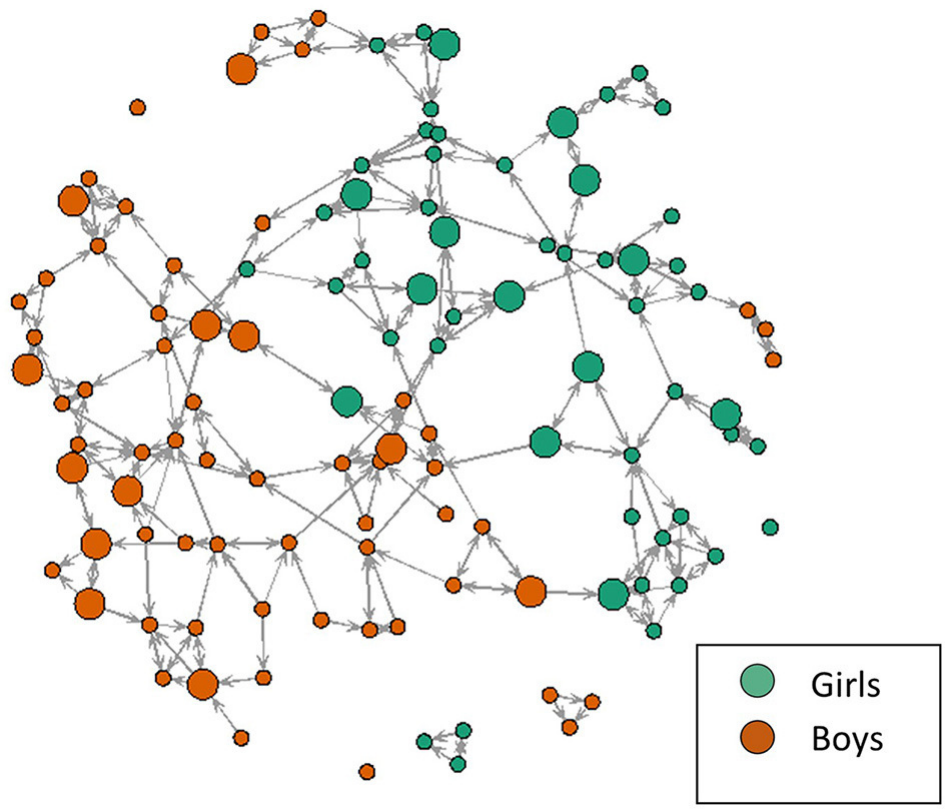
Visual representation of a social network in one school. This figure illustrates the rich information provided by the network: girls are represented in green and boys in orange, social connections are represented by arrows (double arrows if friendships are reciprocated), and larger nodes indicate an insomnia severity index score >9 (Chung et al., 2011).

Adolescents were asked to nominate up to three friends in school. “Outdegree” refers to the nominations given and “indegree” to the nominations received. In this study we will refer to “indegree” as “popularity.” “Betweenness” indicates the extent to which an adolescent in the network bridges separate groups of peers, making him/her the common link between separate cliques and is therefore a measure of centrality within the network (Neal, 2020). Similar to popularity, we hypothesize this role in the network might be linked to both benefits (social connectedness) and pitfalls (e.g., time to be divided among several friend groups). Finally, adolescents who did not have outgoing or incoming nominations in school were defined as “isolates.” Figure 1 illustrates how the data is represented in a social network (i.e., one school), this figure shows the complexity and rich information that can be derived from a network.

#### 2.2.5 Control variables

Depressive symptoms during the last week were measured through the Center for Epidemiological Studies Depression Scale, with 20 items ranging in scores from 0-“Never” to 3-“Always,” and a total score of 60 (CES-D; Radloff, 1977). The scale showed good reliability, α = 0.90. General anxiety during the last week was assessed through the Overall Anxiety Severity and Impairment Scale (OASIS) (Norman et al., 2006). OASIS has 5-items with responses ranging from 0 -“None” to 4 -“All the time.” In this study, the OASIS showed an alpha coefficient of 0.88, indicating good reliability. Alcohol Misuse was measured with one item about getting drunk during the past 6 months, with responses ranging from 1 -“Never” to 5 -“More than 10 times.”

### 2.3 Data analyses

We used R to calculate outgoing nominations (M = 2.3, SD = 0.95, range 0–3) and incoming nominations or popularity (M = 2.3, SD = 1.5, range 0–9) (see Figure 1 for a visual representation of the data). Linear regressions examined the association between popularity and sleep. We ran separate models for insomnia and sleep duration, controlling for confounding variables (demographics, depression, anxiety, and alcohol misuse). Finally, we explored sex differences by running the analyses separately for boys and girls. Missing data were handled using Expectation-Maximization in SPSS, version 26.

## 3 Results

Characteristics of the sample are described in Table 1.

**Table 1:** Descriptives of the study variables in the sample and for boys/girls separately.

	**Total sample (*N* = 1, 395)**	**Boys (*N =* 749)**	**Girls (*N =* 645)**
**Variable**	**Mean (SD)/%**	**Mean (SD)/%**	**Mean (SD)/%**
**Demographics**			
SES	5.88 (1.58)	5.85 (1.62)	5.92 (1.51)
Immigrant background	25%	24.6%	25.6%
**Social network**			
Outdegree	2.25 (0.97)	2.22 (1.04)	2.28 (0.88)
Indegree (Popularity)	2.25 (1.47)	2.22 (1.57)	2.28 (1.35)
Betweenness	144.61 (313.11)	144.24 (310.44)	145.27 (316.62)
Isolates	5.2%	3.6%	6.5%
**Control variables**			
Depression	14.58 (10.93)	11.19 (8.67)	18.49 (11.94)
Anxiety	8.73 (4.06)	7.46 (3.19)	10.20 (4.45)
Alcohol misuse	1.43 (0.98)	1.37 (0.95)	1.50 (1.01)
**Sleep**			
Insomnia symptoms	6.26 (5.58)	5.15 (5.02)	7.55 (5.93)
Sleep duration	7:46 (1:11)	7:55 (1:09)	7:34 (1:11)

After controlling for demographic characteristics and control variables (i.e., depression, anxiety, alcohol misuse), for the whole sample, popularity was associated with shorter sleep duration (B = −3.00; 95% CI [−5.77, −0.19]) but not insomnia symptoms. When analyzing the data separately for boys and girls, popularity was significantly associated with more symptoms of insomnia (Table 3) in girls, but not in boys. For girls, each peer nomination meant an average increase of 0.36 points on the ISI, amounting to 3.2 additional points for the most popular girls. None of the other social network variables were significantly associated with sleep.

**Table 2:** Linear regression models estimating sleep duration (in minutes) in boys vs. girls.

	**Boys**				**Girls**			
	**Model 1**		**Model 2**		**Model 1**		**Model 2**	
**Variables**	**B**	**95% CI**	**B**	**95% CI**	**B**	**95% CI**	**B**	**95% CI**
**Demographic**								
SES	2.68	[−0.57, 5.92]	1.49	[−1.63, 4.62]	0.45	[−3.32, 4.22]	−0.50	[−4.11, 3.12]
Immigrant background	13.58	[0.80, 26.35]^*^	13.04	[0.85, 25.22]^*^	13.20	[0.04, 26.36]^*^	3.53	[−9.49, −16.55]
**Social network**								
Outdegree	9.35	[3.16, 15.53]^**^	4.85	[−1.14, 10.84]	5.66	[−2.57, 13.89]	3.81	[−4.07, 11.68]
Popularity	−1.52	[−5.18, 2.14]	−1.95	[−5.44, 1.54]	−3.48	[−8.33, 1.37]	−4.41	[−9.05, 0.23]
Betweenness	−0.01	[−0.02, 0.01]	−0.01	[−0.02, 0.01]	−0.01	[−0.03, 0.01]	−0.01	[−0.02, 0.01]
Isolate	7.65	[−20.77, 36.06]	−0.94	[−28.10, 26.21]	14.76	[−22.41, 51.93]	17.20	[−18.29, 52.68]
**Control Variables**								
Depression			−2.02	[−2.73, −1.31]^***^			−1.76	[−2.41, −1.12]^***^
Anxiety			1.48	[−0.42, 3.38]			−0.11	[−1.85, 1.62]
Alcohol			−13.42	[−18.57, −8.27]^***^			−2.69	[−8.07, 2.69]

Model 1 includes sociodemographic variables (SES and immigrant background), network variables (outdegree, popularity betweenness, isolate); Model 2 includes mental health controls associated with sleep (depression, anxiety, alcohol misuse). Adjusted ${\text{R}}_{\text{boys}}^{2}$ = 0.10 and ${\text{R}}_{\text{girls}}^{2}$ = 0.09 for the final model. ^*^p < 0.05, ^**^p < 0.01, ^***^p < 0.001.

**Table 3:** Linear regression models estimating insomnia symptoms in boys vs. girls.

	**Boys**				**Girls**			
	**Model 1**		**Model 2**		**Model 1**		**Model 2**	
**Variables**	**B**	**95% CI**	**B**	**95% CI**	**B**	**95% CI**	**B**	**95% CI**
**Demographic**								
SES	−0.36	[−0.60, −0.13]^**^	−0.15	[−0.34, 0.05]	−0.26	[−0.57, 0.06]	−0.08	[−0.33, 0.17]
Immigrant background	0.15	[−0.78, 1.08]	0.13	[−0.64, 0.89]	−0.95	[−2.06, 0.15]	0.75	[−0.15, 1.66]
**Social network**								
Outdegree	−0.06	[−0.51, 0.39]	0.36	[−0.02, 0.73]	−0.31	[−1.01, 0.38]	−0.00	[−0.55, 0.55]
Popularity	−0.13	[−0.39, 0.14]	−0.10	[−0.32, 0.12]	0.21	[−0.20, 0.61]	0.36	[0.04, 0.68]^*^
Betweenness	0.00	[−0.00, 0.00]	0.00	[−0.00, 0.00]	0.00	[−0.00, 0.00]	0.00	[−0.00, 0.00]
Isolate	−0.74	[−2.81, 1.33]	0.27	[−1.43, 1.97]	1.81	[−1.32, 4.94]	1.39	[−1.08, 3.87]
**Control Variables**								
Depression			0.23	[0.19, 0.28]^***^			0.26	[0.22, 0.31]^***^
Anxiety			0.28	[0.16, 0.40]^***^			0.14	[0.02, 0.26]^*^
Alcohol			0.78	[0.45, 1.10]^***^			0.50	[0.13, 0.88]^**^

Model 1 includes sociodemographic variables (SES and immigrant background), network variables (outdegree, popularity, betweenness, isolate); Model 2 includes mental health controls associated with sleep (depression, anxiety, alcohol misuse). Adjusted ${\text{R}}_{\text{boys}}^{2}$ = 0.34 and ${\text{R}}_{\text{girls}}^{2}$ = 0.38 for the final model. ^*^p < 0.05, ^**^p < 0.01, ^***^p < 0.001.

## 4 Discussion

The aim of this study was to investigate how social connectedness among adolescents is associated with their sleep. We found that more popular peers reported shorter sleep duration – up to ~30 min for the most popular peers. When splitting the sample though, we found that popular girls experienced more insomnia symptoms, but not boys.

These results are somewhat in contrast with previous studies in that we did not confirm that being an “isolate” was associated with sleep problems (Li et al., 2019; Palmer et al., 2020), nor that centrality in the network played an important role for adolescents' sleep (Mednick et al., 2010). Instead, receiving many nominations (i.e., popularity) was associated with both shorter sleep and insomnia symptoms, confirming the findings of Li et al. (2019). Interestingly, in Li et al. (2019), data were collected pre-smartphones (1994–95) whereas the present data were collected in 2016 - and yet popular adolescents show similar sleep patterns in these two studies across the decades. This suggests that mechanisms other than the rise of technology use may explain why popular adolescents sleep worse.

Popular girls were more likely to suffer from insomnia symptoms. Gender socialization processes may offer a possible explanation for this, as boys and girls have been found to engage in different friendship behaviors (Rose and Asher, 2017). For example, girls express more care and concern, and engage in helping behaviors more as compared to boys (Rose and Asher, 2017), which might mean they carry these concerns at bedtime and thus have difficulty falling asleep (De Zambotti et al., 2018). The difference in the popularity-sleep association between boys and girls confirms the findings of Li et al. (2019), although we did not find a significant association between being an “isolate” and sleep in boys. Replicating these results in the Swedish context is important, given that differences in sleep exist between Swedish and US adolescents. Swedish girls have been found to sleep less than boys, whereas the opposite is true for US adolescents (see Gariepy et al., 2020), which suggests cultural influences rather than biological changes may be at play. Given only three studies to date have explored gender differences in the link between sleep and popularity, further research is needed to understand potential mechanisms at work.

The results of this study should be interpreted in light of some limitations. Sleep variables were based on adolescents' self-report and not direct measures (e.g., actigraphy). Worth noting is that self-report measures have been found to be accurate in adolescents (Wolfson et al., 2003) and more practical in large samples (>1,000), and actigraphy is both limited in its measurement of insomnia (Buysse et al., 2006) and has been suggested to under-estimate sleep duration (as it is a direct measure of motor activity; Meltzer et al., 2019). Future studies should also consider the contribution of chronotype, as late chronotypes might experience difficulties falling asleep, delay their bedtimes and fill the time with more peer contact. Another caution is that peer nominations were limited to 3 friends, which might be an important difference from the previous studies. Rather than representing peer connections in school, our data might represent “close friends” in this context. On the other hand, popular peers received up to 9 nominations and could be identified even with limited nominations. Moreover, we did not measure time spent on extracurricular activities. Popular adolescents might engage in more activities after school (e.g., sports, work, youth organizations) that reduce their sleep opportunity. Yet, previous studies show only weak associations between sleep duration and time spent in extracurricular activities (Short et al., 2013). Finally, another important limitation is the use of a cross-sectional design to study a phenomenon that may be bi-directional in nature. Social connectedness might affect sleep both positively and negatively, but how we sleep has also shown to affect how we behave in social situations and how well we connect with others (Gordon et al., 2021).

In conclusion, the results of this study suggest that social connectedness is an important area to explore, with the potential to improve our understanding of adolescents' sleep behaviors, in line with other health behaviors (Montgomery et al., 2019). For example, peers' involvement and discussing social norms might be a crucial component of sleep interventions for adolescents. One consistent result thus far is that girls seem to pay a price for their popularity in terms of sleep quality – before and after smartphones became part of adolescents' lives. Future longitudinal studies should examine the possible bi-directionality between social connectedness and sleep, and elucidate the mechanisms explaining discovered sex differences.

## Data availability statement

The raw data supporting the conclusions of this article will be made available by the authors, without undue reservation.

## Ethics statement

The studies involving humans were approved by the Regional Ethical Board in Uppsala, Sweden. The studies were conducted in accordance with the local legislation and institutional requirements. The Ethics Committee/institutional review board waived the requirement of written informed consent for participation from the participants or the participants' legal guardians/next of kin because active consent was obtained from participants and passive consent was obtained from parents to limit sampling bias. That is, parents were asked to send in a form if they did not want their children to participate.

## Author contributions

SB: Writing – original draft, Funding acquisition, Formal analysis, Data curation, Conceptualization. KB: Writing – review & editing, Supervision, Resources, Project administration. MG: Writing – review & editing, Supervision, Conceptualization.
